# Adult pulmonary sequestration: a hidden cause of recurrent hemoptysis (case report)

**DOI:** 10.11604/pamj.2024.48.185.44767

**Published:** 2024-08-27

**Authors:** Amani Kacem, Rahma Ben Jazia, Imen Kharrat, Sami Blel, Anis Maatallah, Jihene Ayachi, Dhouha Ben Braiek, Hend Zorgati, Yosri Ben Ali, Asma Gaddour, Sana Aissa

**Affiliations:** 1 Pneumology Department, University Hospital Ibn Jazzar, Kairouan, Tunisia,; 2 Intensive Care Unit, University Hospital Ibn Jazzar, Kairouan, Tunisia,; 3 Department of Occupational Medicine, University Hospital Ibn Jazzar, Kairouan, Tunisia,; 4 Pneumology Department, University Hospital Farhat Hached, Sousse, Tunisia

**Keywords:** Pulmonary sequestration, hemoptysis, case report

## Abstract

Pulmonary sequestration (PS) is a rare congenital malformation where abnormal lung tissue lacks a connection to the airways and receives blood supply from systemic arteries. This case report describes a 30-year-old man with recurrent hemoptysis diagnosed with intralobar PS (ILS) as the cause. He underwent video-assisted thoracoscopic surgery to clip and section the aberrant artery, resulting in a successful outcome with no further bleeding. The case emphasizes considering ILS in diagnosing hemoptysis, even in adults with atypical presentations, and highlights the utility of CT angiography in identifying the abnormal blood supply for definitive diagnosis and guiding minimally invasive surgical treatment.

## Introduction

Pulmonary sequestration (PS) is a rare congenital lung malformation characterized by abnormal lung tissue that does not communicate with tracheobronchial tree and which receive an abnormal systematic vascular supply [1]. According to Pryce classification, and depending on the presence or absence of a separate visceral pleura covering the pulmonary parenchyma and veinous drainage, PS is divided into extra lobar sequestration (ELS) and intra lobar sequestration (ILS) which is the more common type [2]. The lesion is typically nonfunctional and the presence of abnormal blood supply may cause various complications such as recurrent pneumonia massive hemoptysis and pleural effusion [3]. We report a case of ILS diagnosed in adulthood following the occurrence of recurrent hemoptysis.

## Patient and observation

**Patient information:** we report the case of a 30-year-old male patient who was admitted to the hospital with recurrent low-intensity hemoptysis. There was no associated dyspnea, wheezing, cough, fever or chills. He reported recurrent episodes of similar hemoptysis over the past year, which he had neglected.

**Clinical findings:** the patient had no fever with a temperature of 37.4°C. Auscultation revealed clear breath sounds bilaterally, with no abnormal crepitations. The patient's oxygen saturation was 98%. The patient's hemodynamic status was stable, with normal blood pressure, heart rate, and respiratory rate.

**Figure 1:**
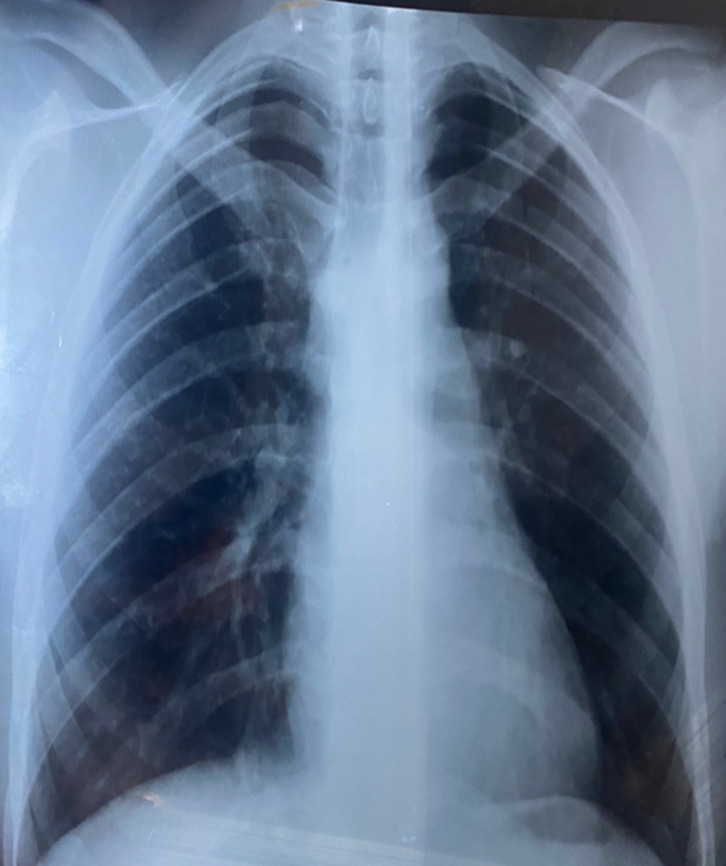
a chest X-ray showing a well-limited paracardial density of 1 cm

**Figure 2:**
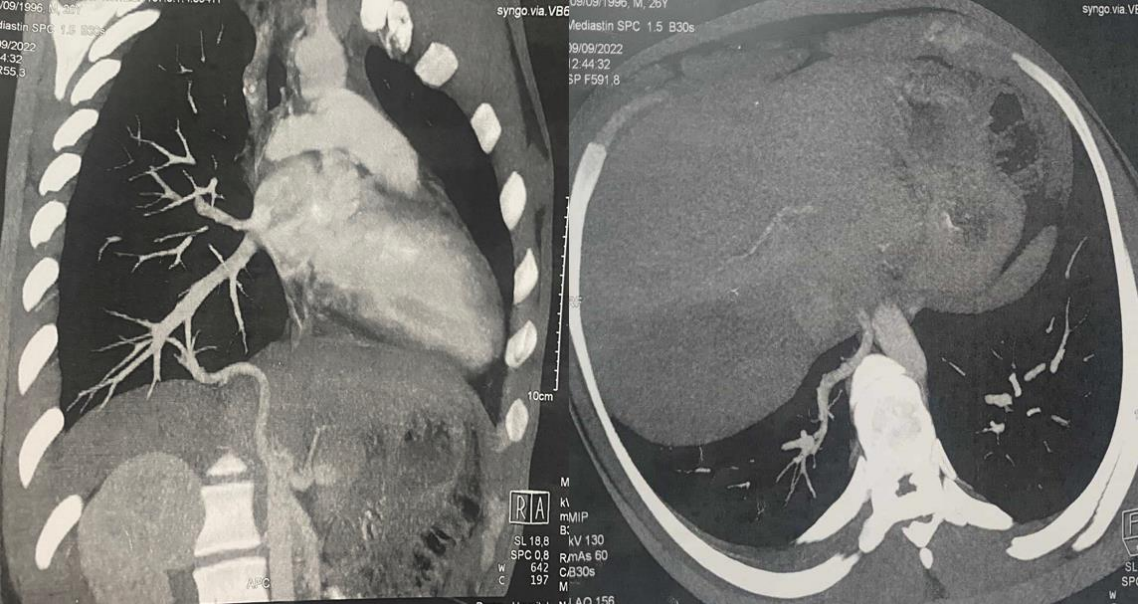
CT angiography showing a 13 mm right posterobasal nodule vascularized by an arterial vessel arising from the subdiaphragmatic aorta above the origin of the celiac trunk

**Diagnostic assessment:** a chest X-ray revealed a well-limited paracardial density of 1 cm (Figure 1). CT angiography showed a right posterobasal nodule measuring 13 mm vascularized by a supplying artery arising from the abdominal aorta above the coeliac trunk (Figure 2). The diagnosis of pulmonary sequestration type I Pryce was made.

**Therapeutic interventions:** the patient was referred to the thoracic surgery department where he underwent a clipping and sectioning of the aberrant artery by video-assisted thoracoscopic surgery.

**Follow-up and outcome of intervention:** the post-operative course was favorable and the patient was discharged from the hospital after 7 days. the long-term outcome was favorable, with no recurrence of hemoptysis.

**Patient perspective:** the patient thinks he received the appropriate treatment and reports no symptoms.

**Informed consent:** the patient gives informed consent for publication.

## Discussion

Pulmonary sequestration (PS) is an uncommon congenital lung malformation characterized by abnormal lung tissue lacking communication with the tracheobronchial tree and receiving its blood supply from the systemic circulation [1]. While typically diagnosed in childhood, its presentation in adulthood, as seen in our case, underscores the importance of considering PS in the differential diagnosis of hemoptysis, even in the absence of other respiratory symptoms. Ps is rare with a prevalence of 0.0225 to 0.425%. [4]. ILS is the most common form of sequestration, accounting for 75 to 86% of all cases. It is more common in adulthood, but in the majority of cases it is diagnosed before the age of 20 [2]. On the other hand, ELS often manifests during the first 6 months of life. this form is associated in 60% of cases with a coexisting congenital anomaly including congenital heart disease, congenital cystic adenomatoid malformation, chest wall and vertebra-diaphragm abnormalities [5].

Our patient presented with recurrent low-intensity hemoptysis, a common manifestation of ILS. However, it is essential to recognize that PS can present with a wide spectrum of symptoms, including recurrent pneumonia, chest pain, or even asymptomatic findings [1]. The atypical presentation in adulthood highlights the diagnostic challenge. CT angiography can reveal several types of lesions, the most common of which are: tissue shadow, cystic lesions, cavitary lesions, bronchiectasis or solid lesions [6]. The most frequent location of lesions is on the right side of the thorax, with a predominance of ILS in the posterobasal regions of the lower lobes [7]. The diagnosis of PS relies on the detection of abnormal artery supply for the sequestrated lung tissue [8]. The most frequent origin of this abnormal vascularization is the thoracic aorta but arteries can sometimes arise from other vessels, including the abdominal aorta, intercostal artery or internal mammary artery. Venous drainage is generally to the pulmonary vessels, but drainage may be to the superior and inferior vena cava, the brachiocephalic vein or the azygos vein [9].

Surgical resection remains the definitive treatment for ILS, aiming to eliminate the abnormal lung tissue and prevent complications such as recurrent infections and hemoptysis. The treatment consists of surgical resection of the abnormal tissue with a preference for minimally invasive surgery using video-assisted thoracoscopy, as employed in our case. The crucial step of the surgical procedure remains the identification and control of the aberrant vascular supply [5]. This treatment not only removes the lesion and prevents complications, but also confirms the diagnosis.

Endovascular embolization has recently emerged as a therapeutic alternative. It reduces blood flow to the sequestrated tissue, leading to necrosis and progressive involution. However, it is still associated with a significant rate of recurrence. This is why it is particularly useful pre-operatively, to minimize the risk of bleeding in patients prone to hemorrhagic complications during surgery [10]. While ILS is relatively rare, its impact on patients can be significant. Our case highlights the importance of considering ILS in the differential diagnosis of hemoptysis, especially in cases with atypical presentations or when other etiologies are excluded.

## Conclusion

This case of adult pulmonary sequestration presenting with recurrent hemoptysis underscores the importance of including PS in the differential diagnosis for this symptom, even without additional respiratory complaints. Our case presentation highlights the diagnostic utility of CT angiography in identifying the anomalous arterial supply, a key feature of PS. Mini-invasive surgical treatment offers a safe and effective treatment approach, leading to symptom resolution and preventing potential complications.
